# Using Appetitive Motivation to Train Mice for Spatial Learning in the Barnes Maze

**DOI:** 10.1155/2023/6625491

**Published:** 2023-12-19

**Authors:** Brigitta Tekla Tajti, Ojin Yoon, Aliz Judit Ernyey, Attila Gáspár, Bence Tamás Varga, István Gyertyán

**Affiliations:** ^1^Department of Pharmacology and Pharmacotherapy, Semmelweis University, Budapest, Hungary; ^2^Doctoral School of Biology and Institute of Biology, Eötvös Loránd University, Budapest, Hungary

## Abstract

The Barnes maze, a well-known spatial-learning paradigm, is based on the innate fear of rodents of large open spaces and their drive to hide. However, additional aversive stimuli (strong light and threatening sounds) are often necessary to provoke the hiding response while rendering the method cumbersome and more stressful. Our objective was to establish a Barnes maze-learning paradigm in mice using palatable food as a reward. After habituating male C57BL6/J or NMRI mice to the reward, the experimenter and the apparatus, either a slow (2 trials/day) or a massive conditioning schedule (4 trials/day), was run. Acquisition training was carried out until mice could locate the reward box with a maximum of one hole error. Then, the box was replaced to another location (reversal phase). Mice needed to relearn the new position with the same criterion. One week later, retention trials were performed. Both strains could reach the learning criteria; in the massive training within a shorter period. Spatial memory was demonstrated in the reversal and retention trials. Our results show that palatable food can be used as an efficient motivator to acquire allocentric navigation in the Barnes maze with the additional advantage of being less stressful.

## 1. Introduction

The Barnes maze is a dry-land navigational model consisting of an elevated circular platform with holes along the perimeter. Underneath one of the holes (target hole) is a box, so called “escape box” or “target box,” through which a test subject can enter. There are extra-maze cues placed around the platform at the level visible from the platform surface. The test subject is put in the middle of the platform, and as rodents have innate tendencies to escape from an environment that they regard as dangerous and have preferences to darkness, the rodent searches for escape possibilities, explores the maze, and enters the target hole that leads to the escape box. Visuospatial memory is assessed by evaluating the ability of a subject to learn and remember the location of the escape box using extra-maze cues.

The Barnes maze was originally developed for testing rats [1], but mice are also tested [2] with alterations like a smaller diameter of platform and longer protocol in general [3]. The diameter of the platform can vary, but the study on the sensitivity of the Barnes maze with different platform diameters for assessing visuospatial learning and memory showed higher sensitivity with a larger diameter. That is, a larger diameter allows mice to utilize more of distant cues and spatial search strategy and less of proximal cues and serial search strategy [4].

The original version of the Barnes maze was performed in a large television studio and used an open-space platform and a bright overhead light to motivate rats to navigate and find the escape box placed beneath one of the holes [5]. The original setup did not use any other external stimuli, as placing the rodent in the middle of an open, brightly lit space itself acts as the motivational factor. The Barnes maze has the advantage of being less stressful than other commonly used spatial learning and memory assays like the Morris water maze and radial arm maze as it does not require swimming in water or long food restriction. On the other hand, its potential drawback is that bright light itself as an aversive stimulus may not be enough to drive mice to perform the experiment [6]. To overcome this drawback, other aversive stimuli, for example, white noise, buzzer, and/or fans, are often added to enhance the motivation of rodents to escape and search for the escape box [6–10].

In our lab, we began to use the Barnes maze with C57BL/6J mice and faced the same difficulty; that is, they showed a lack of motivation to find and enter into the escape box under the aversive conditions we could create. The maximal light intensity was 20 lux in our experimental room, and it was not aversive enough to motivate the mice to escape. We tried to increase the aversiveness by providing loud white noise or playing a dog-barking sound, but they were not successful. Therefore, we chose to provide appetitive stimuli instead of aversive stimuli to instigate the animals to perform the task. Our objective was to establish an appetitively motivated paradigm in the Barnes maze with highly palatable food (chocolate cereals) in mice. In this way, animals may be adequately motivated without being too stressed; hence, confounding effects of stress on performance may be avoided. This is a more animal-friendly version of the Barnes maze that may assess spatial learning and memory as sensitively as using aversive stimuli.

Appetitively motivated Barnes maze protocols in mice are scarce in the literature, and most of them use water as the reward [11–13]. Youn et al. [14] applied food reward, but they used a special version of the Barnes maze with 44 holes scattered around the surface of the maze. Food-motivated protocols for rats were published [15, 16].

We established a food-motivated task with a slow and a massive training protocol. In the former, we compared the performance of two mouse strains (NMRI and C57BL/6); in the latter, we compared releasing the mouse from a start box or the hand of the experimenter. We chose these mouse strains because C57BL6/J and MRI mice are commonly used in the literature, and there are some studies comparing their navigational-learning capabilities in the Morris water maze [17–20]; however, no direct comparison has been made in the Barnes maze so far.

## 2. Methods

We performed 3 experiments. The first two experiments involved a slow training protocol with NMRI mice (Exp1) or with C57BL/6 mice (Exp2). NMRI mice were bred in our lab, while C57BL/6J mice were obtained from Janvier Labs, Le Genest-Saint-Isle, France. During slow training, the animals had 2 trials a day. In the third experiment, a massive training protocol was used with NMRI mice (Exp3) where the mice had 4 trials a day.

### 2.1. Animals

Subjects of Exp1 and Exp3 were six-week-old male Win:NMRI mice (*n* = 12 and *n* = 16, respectively), while in Exp2, twelve 6-week-old male C57BL/6J mice were used. Animals were housed in groups of four in polycarbonate cages (365 × 207 × 140 mm). The holding room had a 12 : 12-hour light-dark cycle, with lights on at 6 : 00 am. Room temperature was 21-23°C and humidity 50-70%. Animals had restricted food access (commercial pellet rat feed R/M–Z + H produced by SSniff) to prevent *ad lib* feeding-induced obesity; the amount of the food was 8 g per cage given at 4 pm. In Exp3, before the retention trials were started (see 2.3. Procedure), mice had an intracage fight in two cages at night and 2 + 2 of them were killed by their cage-mates. Housing and all procedures carried out on animals were authorized by the regional animal health authority in Hungary (Pest County Government Office, resolution number PE/EA/785-5/2019) and conformed to the Hungarian welfare legislation, the EU 63/2010 Directive, and ARRIVE guidelines.

### 2.2. Barnes Maze Apparatus

The Barnes maze (from Ugo-Basile) was a 1 m diameter-wide circular blue metal platform with evenly spaced 20 holes (diameter = 5 cm) along the perimeter. The holes were numbered from 1 to 20 in clockwise direction. The distance between 2 adjacent holes was 14.5 cm along the perimeter or 18-degree viewing from the center. The “reward box” was a small black plastic chamber with a magnetic top by which it could be attached to the under-surface of the platform. Stairs were placed inside to aid mice to enter into it smoothly. It also contained a piece of chocolate cereal which served as the reward for the mice when they found the target hole and entered the box. This reward box was then placed under one of the holes (target hole). To prevent odor-guided navigation, the same cereals were stuck next to each hole beneath the platform.

The Barnes maze was placed in a white-walled moderately lit room (20 lx intensity on the surface level of the maze). In Exp1, the extra-maze cues aiding navigation were black-and-white-patterned cardboards on the wall, a cupboard in the corner, and the door. In Exp2 and Exp3, additional cues were introduced: a stand in another corner and an aluminum foil on the wall.

A video camera was placed above the maze. The experimenter observed the trials on a computer placed outside the experimental room. All sessions were recorded and analysed by Smart v3.0 video-tracking system software (Panlab, Spain).

### 2.3. Procedure

#### 2.3.1. Habituation

The experimental procedure started with habituating the mice to chocolate cereal. Pieces of cereal were placed into the home cage for two nights. Mice ate the chocolate cereal by the next morning. At the same time, all animals were handled by the experimenter for 15 minutes a day, for 2 days.

The next step was habituating the mice to the reward box: we put the cereal and the mice in the reward box and placed it in the middle of the maze. Habituation took place over 2 days. During this session, each animal was allowed to stay in the box for 20 minutes (Exp1 and Exp2) or 15 minutes (Exp3) on the first day and for 15 minutes on the second day.

#### 2.3.2. Acquisition Phase

The following day after habituation, the training started with two different schedules. In Exp1 and Exp2, the animals were tested in 2 trials a day with 2-hour intervals in between (“slow training”). In Exp3, we ran 4 trials per day with 30-minute intervals between them (“massive training”).

In Exp1 and Exp2, at the start of a trial, mice were placed in the middle of the maze and covered with hands for 5 seconds and then released. In Exp3, we assigned the mice into 2 subgroups: 8 mice were released out of hand as in Exp1 and Exp2, whereas the other 8 mice were released from a nontransparent box.

For each trial, mice were given 5 minutes to explore the platform and find and enter the reward box which contained a piece of chocolate cereal. If they failed to enter the box within 5 minutes, they were gently guided into the box (only 2 mice in the NMRI group in Exp1 needed this help at the first trial). After entering the reward box, the mouse was allowed to stay there for 3 minutes; meanwhile, it could eat the chocolate cereal. After this period or when they occasionally came out from the box (whichever happened earlier), the mice were taken back to their home cage. Between trials, the platform and reward box were cleaned with 10% alcohol to eliminate odor cues left over by the previous mouse.

During a trial, the ID number of the holes visited and the time of the visits were recorded by the observer. The movement tracks of the animals were recorded by Smart Video Tracking Program. From these data, the following parameters were derived: (1) latency to find the target hole, (2) latency to enter the reward box, (3) latency to the first visited hole, (4) first visited hole's distance from the target hole (expressed in angle), (5) number of visited holes (excluding repeated visits to the same hole), (6) number of hole visits (including repeated visits to the same hole; this parameter (minus 1) corresponds to the “error” variable in many of the Barnes maze publications), (7) the distance travelled (cm) until finding the target (with Smart Video Tracking Program), (8) number of omission errors: visiting the target hole but moving along instead of entering it, and (9) decision time of entrance: the time elapsed from initiation of exploration of the target until entering it. The last two variables we took from Popović et al. [21] and used them to characterize the motivational state of the animals.

The acquisition phase of the training went on until the group mean of the distance between the first visited hole and the target hole became equal or less than 18°, i.e., at most, one hole error in locating the target.

#### 2.3.3. Reversal Phase

Once animals reached the above learning criterion, the location of the reward box was changed to another hole being 144° away from the original target hole. Same procedures were followed as in the acquisition phase, and mice had to learn to relocate the new target hole. In the slow training protocol, replacement of the reward box always took place between the first and second trial of the day, while in the massive protocol, changing the target hole happened from one day to another. The reversal phase lasted until reaching the same learning criterion.

#### 2.3.4. Retention Test

One week after the last trial in the reversal phase, mice again underwent two trials in the maze with the same target hole as in the reversal phase. Same procedure was followed as in previous sessions. Mice did not perform any tasks during the one-week interval.

### 2.4. Statistical Evaluation

Group means were calculated for each variable in each trial. Statistical analyses of the obtained learning curves were performed by repeated-measures ANOVA with trials as the repeated measure factor (STATISTICA software, version: 13.5.0.13). In Exp3, for comparison of the two releasing methods, factorial repeated-measures ANOVA was applied. To detect statistically significant differences between trials, the post hoc Duncan test was used. In Exp3, missing data of dead mice in the retention trials were replaced by the group mean of the given trial.

Correlations between the measured parameters were also calculated. Daily means of the given variable from all the three experiments (*n* = 123 data points) were used for the correlation analysis.

## 3. Results

### 3.1. Latency to Find the Target Hole

**Figure 1:**
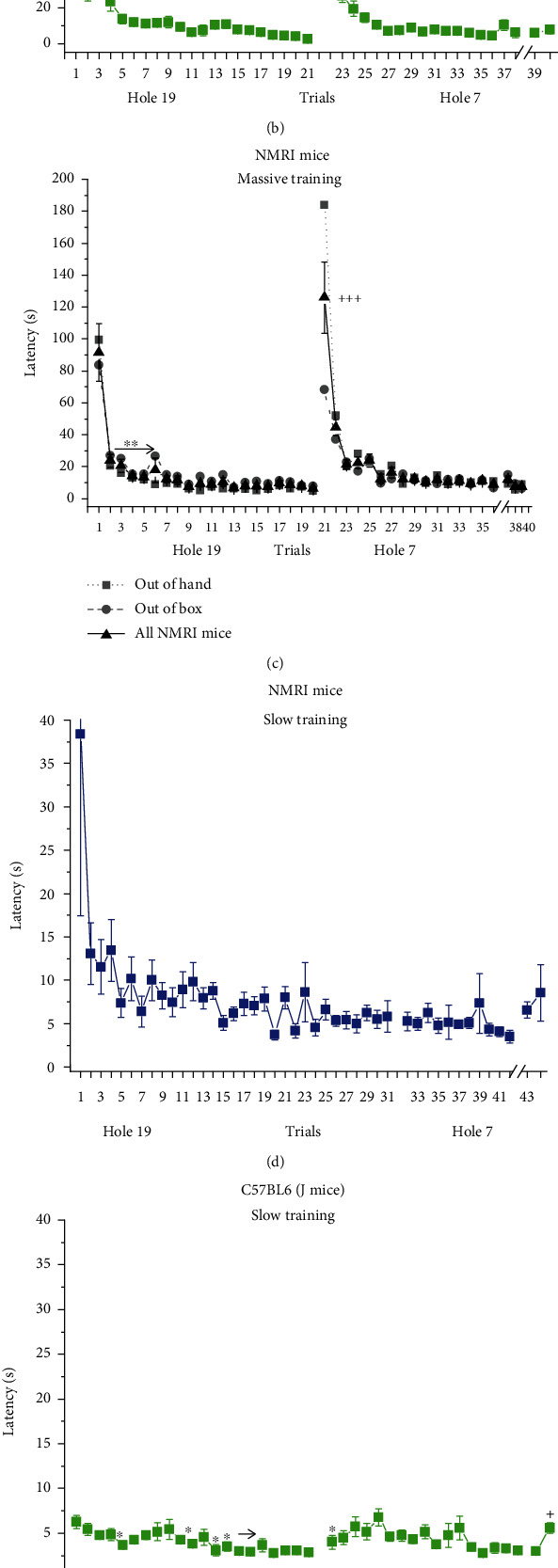
Latency to find the target hole (a–c) and latency to visit the first hole (d–f) in the food-motivated Barnes maze task. Mean ± SEM values are shown. Performance of NMRI (Exp1) (a, d) and C57BL/6 (Exp2) mice (b, e) in the slow protocol. (c, f) Performance of NMRI mice in the massive protocol (Exp3). Black lines and symbols show the pooled data of the two subgroups (“out of hand” and “out of box”). Gray lines and symbols show the data of the two subgroups. S.E.M. bars are omitted from these curves for the sake of clarity. ^∗,∗∗,^∗∗∗^^*p* < 0.05, *p* < 0.01, and *p* < 0.001 vs. 1st trial; ^++,+++^*p* < 0.01 and *p* < 0.001 vs. previous trial. The arrow indicates that the trial and the following trials significantly differ from trial 1.

These results are shown in Figures 1(a)–1(c). In all the three experiments, two phases of the acquisition curves can be distinguished: a steep, rapid decrease in the first 3-5 trials followed by a slowly decreasing, longer lasting, flatter part. Latency to find the target hole dramatically increased at the first reversal trial, and time courses of relearning the new target location were similar to those in the acquisition phase in all experiments. Latencies during retention trials did not significantly differ from the last reversal trial performed a week before.

In Exp1 (ANOVA: *F*(43, 473) = 7.4975 and *p* < 0.001), latency to the target hole in trials from trial 2 significantly differs from the latency on trial 1 (*p* < 0.001). There was no significant difference among trials between trial 4 and trial 31 (the last acquisition trial). Trial 32 (the first reversal trial) shows a significant increase (*p* < 0.001) compared to the previous trial and a significant decrease vs. trial 1 (Figure 1(a)).

In Exp2 (ANOVA: *F*(39,429) = 16.666 and *p* < 0.001), the latency to the target hole was significantly shorter in trial 2 and subsequent trials (*p* < 0.01) compared to trial 1, whereas no significant difference was found among trials between trial 5 and trial 21 (the last acquisition trial). The latency in the first reversal trial significantly increased compared to that in both the previous trial and trial 1 (Figure 1(b)).

In Exp3, no significant difference was found between the 2 groups (“out of hand” and “out of box”) (ANOVA: *F*(1, 14) = 0.446 and *p* > 0.05) while the effect of “trials” was highly significant (ANOVA: *F*(38, 532) = 24.105 and *p* < 0.001). Biphasic curves can also be seen in this experiment: the latency to the target hole was significantly shorter in trial 2 and subsequent trials (*p* < 0.01) compared to trial 1, whereas no significant difference was found among trials between trial 3 and trial 20 (the last acquisition trial). The latency in the first reversal trial significantly increased compared to that in both the previous trial and trial 1 (Figure 1(c)).

### 3.2. Latency to Visit the First Hole

This parameter—in contrast to the latency-to-find variable—did not change much during the whole course of the experiments with the exception of the first trials in Exp1 and Exp3 (Figures 1(d)–1(f)). In Exp1, ANOVA yielded a significant result (*F*(43, 473) = 2.2709 and *p* < 0.001) due to the outstanding value of the very first trial (resulted from an outlier animal having a latency of 287 s), which significantly differed from all the other trials, while the latter did not significantly differ from each other (Figure 1(d)).

In Exp2 (ANOVA: *F*(39, 429) = 2.8345 and *p* < 0.001), the latency ranged between 2.5 and 6 s, and no significant differences were found between trial 21 and trial 22 (i.e., at reversal) and between trial 38 and trial 39 (retention test) (Figure 1(e)).

In Exp3, a significant difference was found between the two groups (ANOVA: *F*(1, 14) = 7.8420 and *p* < 0.05); mice released from the box had on average 2.1 s higher latency than those released from hand. Post hoc test of the repeated-measures factor (ANOVA: *F*(38, 532) = 12.3151 and *p* < 0.001) showed that the latency in trial 1 was significantly higher than all the other values, while no significant difference was detected between trial 20 and 21 (reversal) and between trial 36 and 37 (retention) (Figure 1(f)).

### 3.3. First Hole Distance from Target Hole in Angle

**Figure 2:**
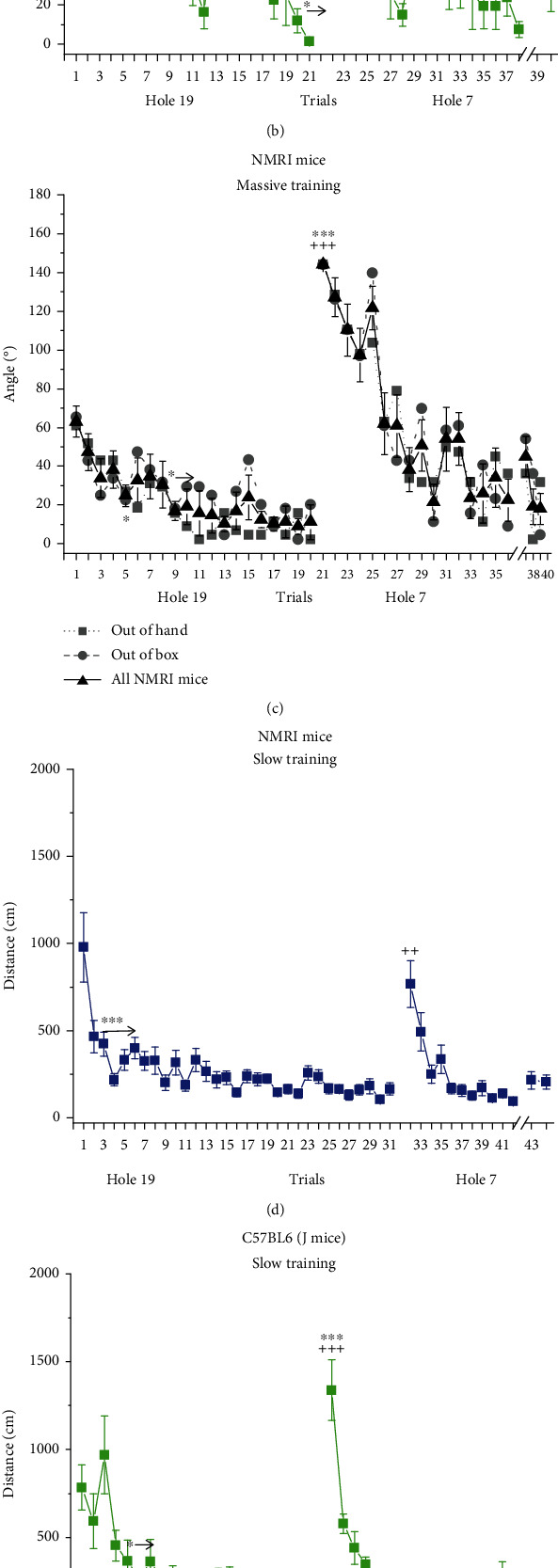
First hole distance from the target hole (a–c) and distance travelled (d–f) in the food-motivated Barnes maze task. Mean ± SEM values are shown. Performance of NMRI (Exp1) (a, d) and C57BL/6 (Exp2) mice (b, e) in the slow protocol. (c, f) Performance of NMRI mice in the massive protocol (Exp3). Black lines and symbols show the pooled data of the two subgroups (“out of hand” and “out of box”). Gray lines and symbols show the data of the two subgroups. S.E.M. bars are omitted from these curves for the sake of clarity. ^∗,∗∗,^∗∗∗^^*p* < 0.05, *p* < 0.01, and *p* < 0.001 vs. 1st trial; ^++,+++^*p* < 0.01 and *p* < 0.001 vs. previous trial. The arrow indicates that the trial and the following trials significantly differ from trial 1.

Change in this variable reflects spatial (navigational) learning (acquisition of navigational strategy) where zero degree indicates precise localization of the target hole. These curves show a different time course from the latency to find or distance travelled curves (Figures 2(a)–2(c)). A common feature of them in all experiments is that the distance at the first reversal trial, which significantly differs from those in both the first and last acquisition trials, is about 144° which corresponds to the position of the target hole in the acquisition phase.

In Exp1 (ANOVA: *F*(43, 473) = 2.8449 and *p* < 0.001), no significant difference could be found between the first trial and any other trial in the acquisition phase. Albeit distance in the retention trials (trial 43 and 44) was higher than in the last reversal trial (trial 42), these differences were not significant (Figure 2(a)).

In Exp2 (ANOVA: *F*(39, 429) = 6.2316 and *p* < 0.001), only the last two acquisition trials showed significantly different values from the first trial. Similar to Exp1, distance in the retention trials (trial 39 and 40) was higher but not significantly different from that in the last reversal trial (trial 38) (Figure 2(b)).

In Exp3, ANOVA revealed no significant group effect and interaction, but significant time effect (ANOVA: *F*(38, 532) = 11.552 and *p* < 0.001). Post hoc test of the latter showed significant difference between trial 1 and trials 5 and 9-20. Again, there was no significant difference between the retention trials (37-39) and the last reversal trial (trial 36) (Figure 2(c)).

### 3.4. Distance Travelled

**Figure 3:**
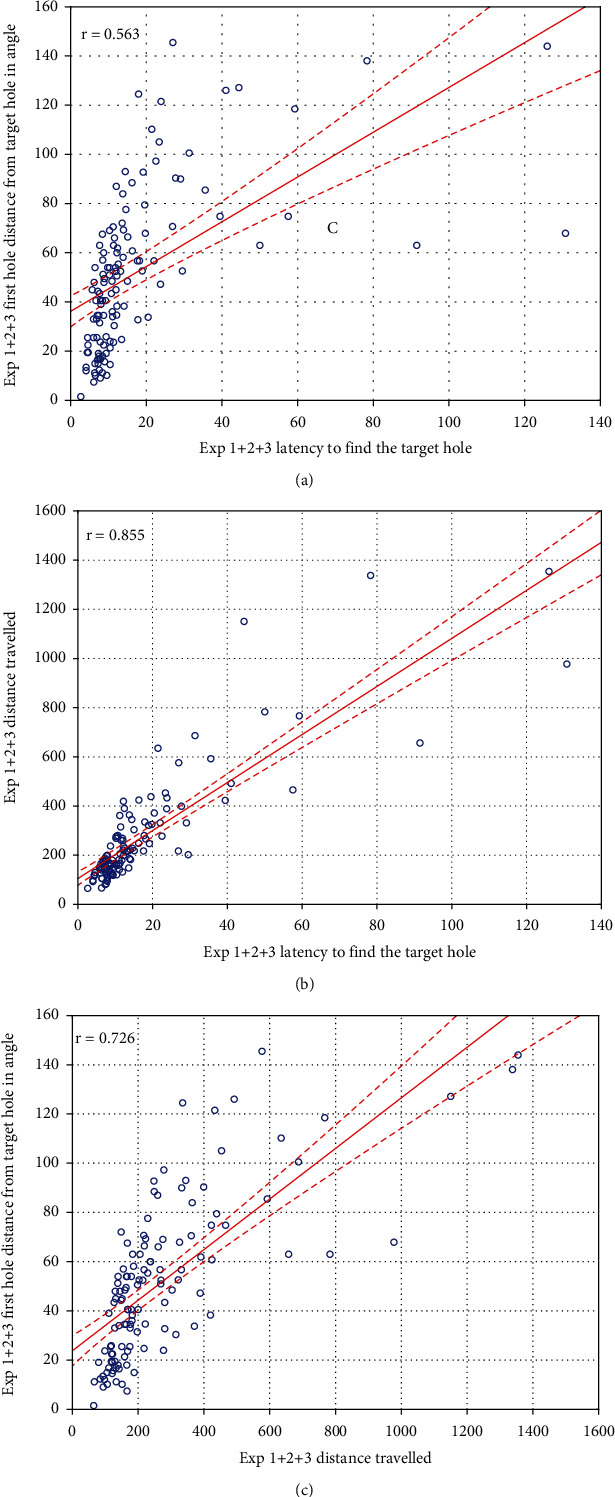
Correlations between measures of learning performance. (a) Latency to find the target hole and first hole distance from the target hole in the angle. (b) Latency to find the target hole and distance travelled. (c) Distance travelled and first hole distance from target hole in the angle. Dotted lines show 95% confidence interval of the regression line. All the 3 *r* values are significant.

These results are shown in Figures 3(d)–3(f). Common features of all the three curves are as follows: (i) distance travelled significantly increased at the first reversal trial compared to the last acquisition trial and (ii) distance during retention trials did not significantly differ from the last reversal trial performed a week before.

In Exp1 (ANOVA: *F*(43, 473) = 7.6181 and *p* < 0.001), a biphasic curve can be seen in the acquisition phase, which runs parallel with the “latency to find” curve. Distance travelled until finding the target hole in trials from trial 2 significantly differed from the distance in trial 1 (*p* < 0.001). There was no significant difference among trials between trial 7 and trial 31 (the last acquisition trial) (Figure 2(d)).

In Exp2 (ANOVA: *F*(39.429) = 12.810 and *p* < 0.001), the decrease of the travelled distance is rather gradual during the acquisition phase. From trial 4, all the trials significantly differ from trial 1, and no significant difference was found among trials between trial 8 and trial 20 (Figure 2(e)).

In Exp3, no significant difference was found between the 2 groups (ANOVA: *F*(1, 14) = 0.087, *p* > 0.05) while the effect of “trials” was highly significant (ANOVA: *F*(38, 532) = 13.675 and *p* < 0.001). Distance travelled until finding the target hole was significantly shorter in trial 2 and subsequent trials (*p* < 0.05) compared to trial 1, whereas no significant difference was found among trials between trial 4 and trial 20 (the last acquisition trial) (Figure 2(f)).

### 3.5. Omission Errors and Decision Time

In Exp1, only one omission error was detected during the whole training period. In Exp2, 12 omission errors occurred (2.5% incidence in all the trials) out of which 9 were committed by 5 mice within the first 5 trials. In Exp3 (massive training), the incidence of omission errors was higher (7.4% and 8.0% in the two groups). About half of them were committed by one animal in each group (responsible for 3.8% and 5.1% incidence) which developed the “habit” of rapidly checking the reward box at the target hole then continuing to explore the maze and then returning to the box to consume the pellet. The decision time of entrance was low and quite uniform in all experiments (3.9 s, 5.0 s, and 4.5 s on average in Exp1, Exp2, and Exp3, respectively). Its change during training is shown in the supplementary file, in Figure S1.

### 3.6. Correlation between the Various Learning Parameters

Data are shown in Figure 3. The latency-to-find and distance-travelled parameters showed the highest correlation (*r* = 0.85), whereas the latency-to-find and distance-from-target-hole variables weakly correlated (*r* = 0.56).

### 3.7. Search Strategies

**Figure 4:**
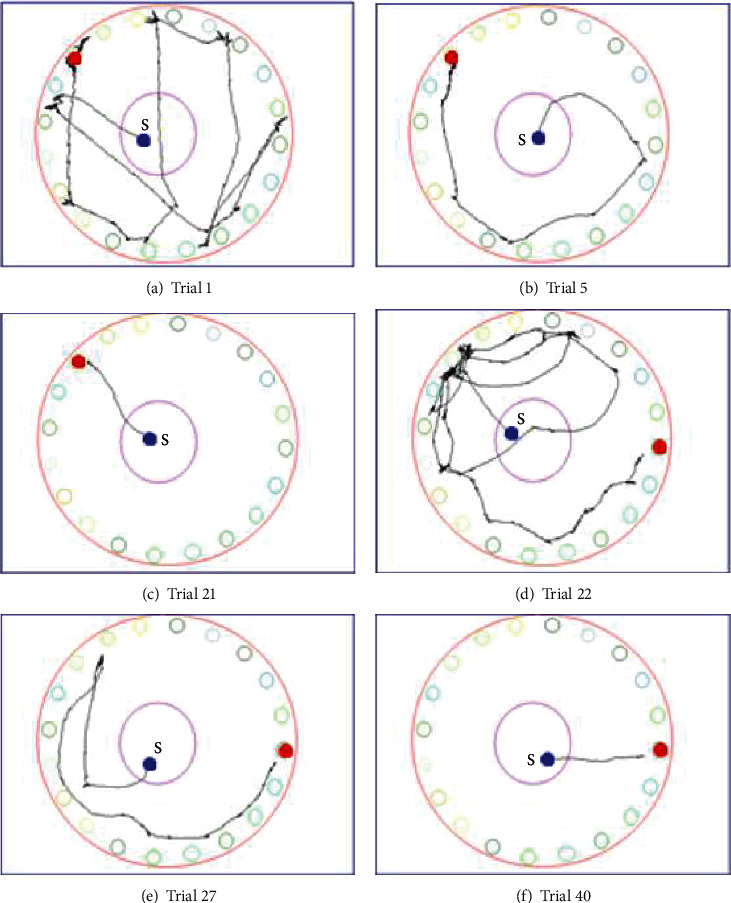
Sample track images showing tracks travelled by mice in Exp2. (a–c) Acquisition phase; (d–f) reversal phase. “S” represents the place where mice started navigation; red dot represents the target hole.

Figure 4 shows track samples of the routes mice travelled during trials in Exp2. On the first trial, mice travelled randomly across the maze and along the perimeter until they finally found the target hole (Figure 4(a)). On trial 5, mice travelled more along the perimeter, visiting holes serially (Figure 4(b)). At the end of the acquisition training, on trial 21, mice immediately headed to the target hole (Figure 4(c)). In the reversal phase, on the first trial after the displacement of the reward box (trial 22), tracks were initially concentrated around the previous target hole. This phase was followed by checking the neighbouring holes with recurrent returning to the former target. Finally, the mouse found the new target location by serial search (Figure 4(d)). On trial 27, the first hole to visit was still the previous target hole, but soon after, the mouse switched to serial search and found the new target location (Figure 4(e)). In the last trial of the reversal training, mice directly ran to the target hole.

## 4. Discussion

In all the 3 experiments, mice successfully learned the location of the target holes with at most a one-hole error, i.e., using spatial navigation as a cognitive tool in the Barnes maze. The animals reached this level without aversive motivation, reflecting the effectiveness of palatable food reward as a motivator.

### 4.1. Biphasic Learning during Acquisition

Comparison of the latency to find curves to initial error data suggests that spatial navigation learning consisted of 2 phases. During the first 3-5 trials, a steep decrease can be seen in the latency to find the target hole reaching a significant level already from trial 2 compared to the first trial. In contrast, first hole distance from the target hole (initial error) did not decrease during this phase (Exp1 and Exp2) or only moderately decreased (Exp3). During this phase, mice learned the context of the task, namely, that the reward box (which they had already been familiar with) was under one of the holes, and they could enter it and consume the reward. Their initial random search strategy (moving across and along the periphery of the maze) progressively and relatively quickly switched to serial search strategy (they ran out to the edge of the platform and then moved around the perimeter until they found the reward box). This transition from random to serial search strategy underlay the steep decrease in the latency to find the reward box. Nevertheless, both random and serial searches are nonspatial strategies that do not utilize distal cues to find the target; thus, their location accuracy is low. In the second phase of learning characterized by a slow, gradual decrease in latency from trials 3 to 5 up to the last acquisition trial, the initial error decreased and reached the level of one-hole accuracy. During this phase, mice learned to use the extra-maze cues to locate the target hole; that is, they switched to the spatial navigation search strategy.

### 4.2. Reversal: The Initial Error Shows That Mice Used Spatial Strategy

Once mice reached our set learning criterion, i.e., when the mean of distance between the first visited hole and the target hole reached equal or less than one-hole deviation, we displaced the reward box to another hole, 8 holes (144°) away from the original target location, and carried out the same procedure as in the acquisition phase. In the first reversal trial, latency to find the target hole, first hole distance from the target hole, and total distance travelled until finding the target hole, but not latency to visit the first hole, showed significant increase compared to the last acquisition trial. Such significant increase resulted from mice first heading to the previous target hole indicated by the first hole distance from the target hole being about 144°. The tracks and the video recordings showed that mice were heavily engaged in recurrently exploring the former target hole while intermittently visiting adjacent holes for long periods. In Exp2 and Exp3, this insistence on the previous target even resulted in significantly greater latency and distance values than those in the very first acquisition trial. These results undoubtedly show that the animals indeed learned to precisely locate the reward box in the first phase of training. Relearning the new location of the target hole involved similar phases as in the acquisition trials: first is a random search (only in the very first trial), then serial search (often already in the first trial), then spatial navigation.

### 4.3. Partial Memory after One-Week Pause

Once mice learned to locate the new target hole with less than one-hole deviation on average, we took a 1-week break in training to assess navigational memory in a retention trial. The performance of mice at these occasions did not significantly differ from that in the last reversal trials in either experiment; nevertheless, their location accuracy somewhat decreased (44-53°). Although the animals could not exactly locate the target hole, they searched it in the right quadrant, suggesting that they still had traces of spatial memory.

### 4.4. Sudden Changes in the Performance of C57BL/6 Mice

In Exp2, presumably owing to the enriched extra-maze cues, C57BL/6 mice showed a faster increase in their navigational accuracy (i.e., a quicker decrease in the first hole distance from the target hole) than NMRI mice did in Exp1. However, sudden impairments can be seen in the performance in trial 13 in the acquisition phase and trial 29 in the reversal phase. C57BL/6 mice partially lost the accuracy they had already acquired reflecting a reduced use of spatial search strategy and more of serial search strategy. These unexpected impairments may be accounted for due to a change in the experimenter. The person who began the experiment got ill, and a new experimenter substituted and took over her job from trial 13. Then, the former experimenter returned on the day of trial 29 and carried on with the remaining experiment. We assume that the change of the experimenter meant a stressful situation for the mice at both occasions, which may have temporarily impaired their performance. This assumption is supported by the findings from Schwabe et al. [22] showing that acute stress induced a shift in search strategy from hippocampus-based spatial-learning strategy to caudate-based stimulus-response-learning strategy in mice.

### 4.5. Massive Training

With the training protocols used in Exp1 and Exp2, Barnes maze learning is a long experiment. In Exp3, we wanted to investigate whether it can be shortened with massive protocol training. The results demonstrate that both the acquisition and reversal phases can take place within a week to reach maximum one-hole deviation in accuracy—a criterion usually not set and not achieved in the literature. In this experiment, we also investigated whether there is any difference between two release methods: (i) releasing the mouse from the hands of the experimenter and (ii) releasing it from a pot. The significant difference found in the latency-to-first-hole variable was due to a short period after lifting the covering pot needed for the mice to look around and recognize where they are. However, we did not find significant difference between the two groups in the essential learning parameters (latency to find, distance travelled, and initial error) or motivational variables (number of omission errors and decision time of entrance) suggesting that both release methods can be used in the Barnes maze. However, the effect of the experimenter change in Exp2 shows that in case of releasing from hand, the experimenter should remain the same person throughout the experiment to prevent the mice's eventual stress reaction.

### 4.6. Comparison with Other Studies Using Appetitive Motivation in the Barnes Maze

There are few papers on an appetitively motivated version of the Barnes maze. Three of them used 22-hour water restriction to motivate animals, which itself renders the task too stressful [11, 12, 23]. Another study on the Barnes maze with appetitive motivation [24] did not provide enough information on how the appetitive motivation was carried out. We found 3 evaluable studies using food reward as an incentive. Youn et al. [14] compared the effects of aversive and appetitive motivations on 2 strains of mice (C57BL/6J and DBA) using a modified Barnes maze. They found that on the aversive version, C57BL/6J mice performed better, but on the appetitive version, both strains were equally competent. However, it has to be noted that Youn et al. [14] utilized the modified Barnes maze that has 44 holes scattered on the platform surface in which the arrangement makes it difficult to clearly define the search strategy of the animals and compare it with our result.

The studies done by Williams et al. [16] and Heimer-McGinn et al. [15] in rats are the most comparable to ours in that they both used the classic Barnes maze and motivated their animals with highly palatable food rewards. They also performed 2 trials per day that is equivalent to our procedure in Exp1 and Exp2. However, Williams et al. only ran the training for 5 days and obtained contrasting results: on day 5, the latency-to-target hole was 190 seconds for male rats and 250 seconds for female rats. Although these values reflect some improvement in performance as the latency was more than 400 seconds on day 1, this improvement does not verify spatial learning as this time may well have been enough for the animals to find the target hole by serial or even random search strategy, compared with our study, where the latency to target was already less than 20 seconds and less than 10 seconds in trial 10 in Exp1 and Exp2, respectively. Furthermore, the latency to the first hole was 30 seconds on day 5 in the rat study, which in our case was 6 seconds in Exp1 and 4 seconds in Exp2. The quite long latencies in this study raise doubt on how motivated the animals were. In our study, the low number of omission errors and the short decision times (beside the short latencies) reflected a high level of motivation. Heimer-McGinn et al. [15] compared socially housed and nonsocially housed Long-Evans rats, and the training in the Barnes maze went on for 8 days. Within this time frame, animals reached short latency to find the target (less than 6 s, which is similar to our values) but mostly relied on the serial search strategy as was emphasized by the authors. Only on the last two days were there signs that some of the rats were switching from serial to direct search.

In conclusion, our results demonstrate that palatable food reward as a motivator was effective in the Barnes maze in mice. Animals learned to locate the target hole with less than one-hole deviation by using genuine allocentric spatial navigation. Spatial memory in mice was demonstrated on reversal and retention trials. Our appetitively motivated paradigm was equally competent for assessing spatial learning and memory in mice compared with the classic Barnes maze protocol regarding the achievable performance level as well as the learning speed (cf. with data from [10, 25, 26]). However, the here presented paradigm bears several advantages: (i) it is less stressful than the aversive version or the water-deprivation protocol; (ii) no need of special equipment like fans, reflectors, and sound sources; (iii) spatial navigation can be acquired within a week (with the massive training protocol); thus, the method is comparable to the Morris water-maze training in terms of time demand. Although prior habituation of the mice to the rewarding box and to the experimenter is time consuming, this step is essential to prevent possible stress reactions of the animals. Besides animal welfare aspects, the less stressful character of the method may prove to be remunerative in longitudinal studies or when the mice are simultaneously tested in several cognitive assays. Further studies are needed to extend the applicability of the method to additional mouse strains.

## Data Availability

The data used to support the findings of this study are available from the corresponding author upon request.

## Supplementary Materials

Supplementary Materials The supplementary files contain results of decision time of entrance as a function of training trials (Figure S1).
