# Supplementary material for: Using Appetitive Motivation to Train Mice for Spatial Learning in the Barnes Maze
Source: Biomed Res Int. 2023 Dec 19;2023:6625491. doi: 10.1155/2023/6625491 (PMC10751176; doi:10.1155/2023/6625491)
Supplement: Supplementary Materials — The supplementary files contain results of decision time of entrance as a function of training trials (Figure S1). [file 6625491.f1.docx]

Supplementary file

*Decisison time of entrance as a function of training trials*

In Exp1 and Exp2 the curves show simalar course to those of the latency to find curves, inasmuch there is a biphasic pattern in the acqusition phase and an abrupt increase at the first reversal trial. The ’trial’ effects were significant in both experiments (F(43, 473)=7.8458, p<0.001 in Exp1 and F(39,429)=7.1709, p<0.001 in Exp2).

In Exp3 neither signficant group effect nor signficant interaction was detected. The effect of ’trial’ was, however, signficant (F(38, 532)=3.233, p<0.001). Post-hoc analysis revelaed that that decisison time in the first reversal trial signficantly differed from that in all other trials.

Figure S1. Decision time of entrance in the food-motivated Barnes maze task. Mean ± SEM values are shown. (A) and (B): performance of NMRI (Exp1) and C57Bl/6 mice (Exp2), respectively in the slow protocol. (C): performance of NMRI mice in the massive protocol (Exp3). Black lines and symbols show the pooled data of the two subgroups (‘out of hand’ and ‘out of box’. Gray lines and symbols show the data of the two subgroups. S.E.M. bars are omitted from these curves for the sake of clarity. *, **, ***: p<0.05; p<0.01, p<0.001 vs. 1st trial; ++, +++: p<0. 01, p<0.001 vs. previous trial. The arrow indicates that the trial and the following trials significantly differ from trial 1.
